# Embedding science in AI for inverse materials design

**DOI:** 10.1093/nsr/nwag615

**Published:** 2026-09-28

**Authors:** Tao Lyu, Jie Xiong, Tong-Yi Zhang

**Affiliations:** Materials Genome Institute, Shanghai University, China; Materials Genome Institute, Shanghai University, China; Materials Genome Institute, Shanghai University, China

Artificial intelligence for science (AI4S) is rapidly reshaping materials discovery, from large-scale computational exploration to experimentally validated inverse design [1,2]. Yet much of this progress still relies on a conventional division of roles, with science providing data and AI providing predictive machinery. This approach can be powerful, but materials problems are rarely unconstrained prediction tasks. Data are sparse and heterogeneous, mechanisms span multiple scales, and practical applications usually require navigating competing objectives [3]. A key question for the next stage of AI4S is therefore not only how AI can advance science, but how scientific knowledge should reshape AI. Scientific knowledge can be embedded into AI through physical constraints, mechanistic representations and physics-aware architectures [4]. Such integration may be particularly valuable when extrapolation, interpretability and multi-objective trade-offs matter as much as predictive accuracy.

Ding, Zou and co-workers provide a compelling demonstration of this emerging paradigm in *National Science Review* [5]. They developed a physics-constrained inverse-design system (PHICS, Fig. 1a) for composite phase-change materials (CPCMs), effectively reconciling the long-standing trade-offs among latent-heat storage, thermal conduction and electrical insulation. While graphitic fillers (GFs) enhance thermal conductivity, they inevitably dilute the phase-change matrix and form electrical pathways. Rather than relying on a black-box mapping from composition to performance, PHICS organizes established multiscale physical relationships into a directed acyclic graph. Interconnected modules capture thermal storage, interfacial thermal resistance, bulk thermal and electrical conductivities, bridging microscopic interactions, mesoscopic networks and macroscopic properties. Crucially, physics enters not as an extrinsic loss penalty, but as the underlying architecture itself, governing the forward information flow. Coupled with a diversity-enhanced NSGA-II optimizer, PHICS efficiently navigates a five-dimensional design space to identify Pareto-optimal compromises across competing functional demands.

**Figure 1.**
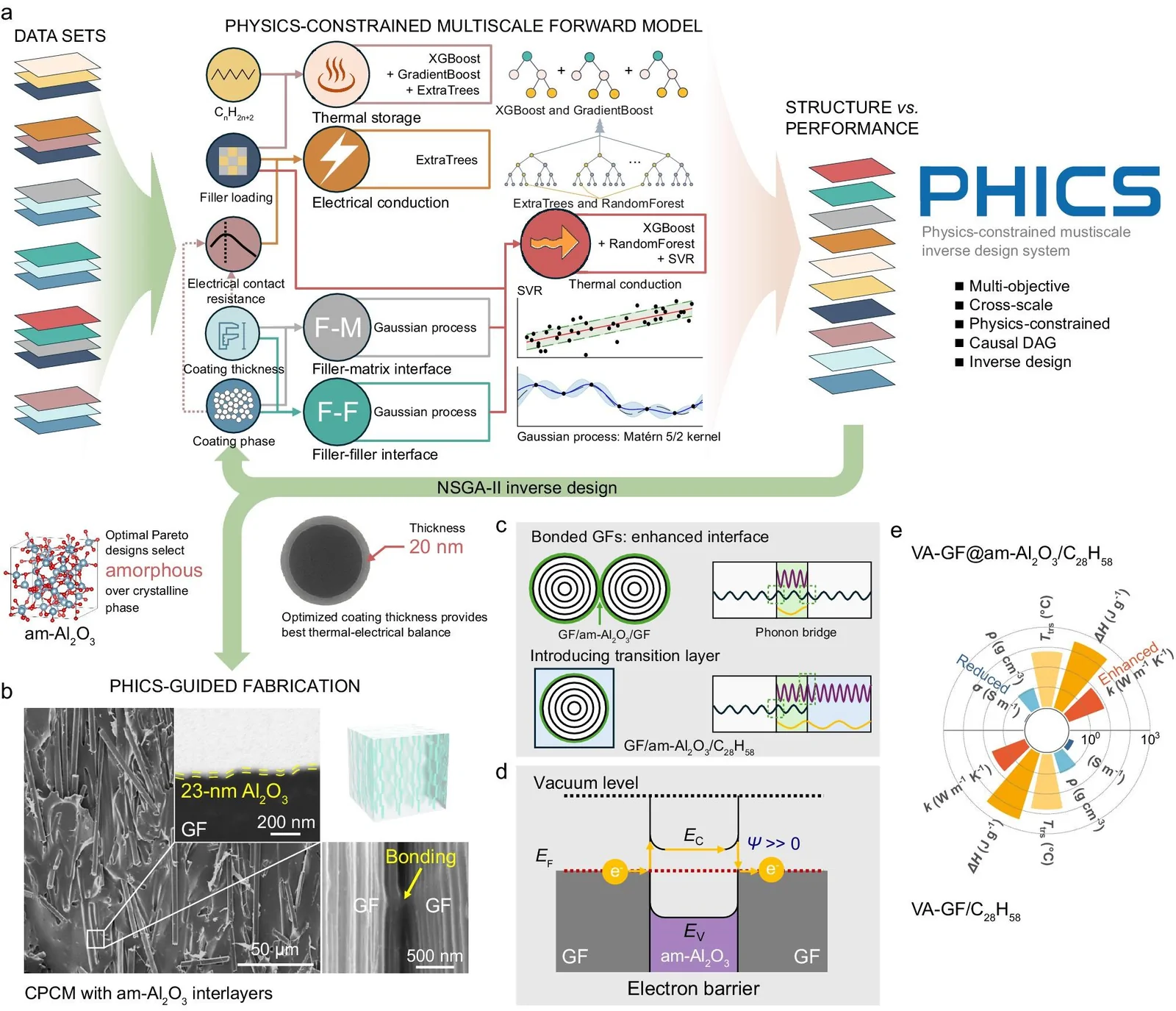
Embedding multiscale physics in AI for inverse CPCM design. (a) Five controllable variables serve as inputs to the multiscale PHICS framework, where physical causality governs model architecture and information flow; NSGA-II is subsequently employed to identify Pareto-optimal trade-offs among thermal storage capacity, thermal conductivity and electrical insulation. (b) The fabricated VA-GF@am-Al_2_O_3_/C_28_H_58_ CPCM integrates a VA-GF, an *n*-octacosane matrix and am-Al_2_O_3_ coating interlayers, playing dual roles as (c) a phonon bridge to enhance thermal transport and (d) an electron barrier to suppress electrical conduction, thereby (e) achieving synergistic enhancements in thermal conductivity and electrical insulation while maintaining superior overall performance. Conceptual illustration adapted from Ref. [5].

The resulting design integrates an *n*-octacosane (C_28_H_58_) matrix and a vertically aligned GF skeleton (VA-GF) with the amorphous alumina (am-Al_2_O_3_) coating (Fig. 1b). Remarkably, the am-Al_2_O_3_ interlayer in the CPCM facilitates an interfacial phonon bridge (Fig. 1c) while simultaneously acting as an electron barrier (Fig. 1d). Guided by the Pareto solutions, the authors fabricated the target CPCM with a 23-nm am-Al_2_O_3_ interlayer, achieving a high thermal-storage capacity of 238 J g^−1^ (25–65°C), an enhanced thermal conductivity of 7.80 W m^−1^ K^−1^ and a suppressed electrical conductivity of 0.093 S m^−1^ (Fig. 1e).

The significance of PHICS extends beyond this high-performance thermal-management material. The framework demonstrates how scientific knowledge can become part of what AI represents and reasons with, rather than serving only as training data or *post-hoc* validation. This principle may provide an important stepping stone toward materials world models. Such models would go beyond composition–property prediction to capture interactions among composition, processing, structure, interfaces and properties. Integration of physical laws, simulations and experiments could further enable them to reason about how materials evolve under different conditions. Their ambition would be not only to predict material states, but also to anticipate how those states would change under hypothetical interventions and explain why. PHICS therefore offers more than an effective inverse-design framework. It provides a glimpse of how AI4S may evolve from property prediction to scientific reasoning and ultimately to models of the materials world.
